# Green ripe fruit in tomato: unraveling the genetic tapestry from cultivated to wild varieties

**DOI:** 10.1093/jxb/erae149

**Published:** 2024-06-07

**Authors:** Pankaj Kumar, Mohammad Irfan

**Affiliations:** Department of Biotechnology, Dr. Y.S. Parmar University of Horticulture and Forestry, Solan, Himachal Pradesh 173230, India; Plant Biology Section, School of Integrative Plant Science, Cornell University, Ithaca, NY, USA

**Keywords:** Fruit domestication, fruit ripening, green ripe fruit, RIN, RNAi, *Solanum habrochaites*, *Solanum pennellii*, wild tomato

## Abstract

This article comments on:

Cui L, Zheng F, Li C, Li G, Ye J, Zhang Y, Wang T, Hong Z, Ye Z, Zhang J. 2024. Defective mutations in *STAY-GREEN 1*, *PHYTOENE SYNTHASE 1*, and *MYB12* genes lead to formation of green ripe fruit in tomato. Journal of Experimental Botany 75, 3322–3336.

**Fruit color, a significant consumer trait, is governed by a complex interplay of pigments and mutations in the carotenoid biosynthesis pathway. The down-regulation of three fruit color-related genes, *SlSGR1*, *SlPSY1*, and *SlMYB12*, results in green fruit at maturity in tomato. Using a green ripe tomato cultivar (Lvbaoshi or Emerald) and several wild tomato species, Cui *et al*. (2024) identified a 603 bp deletion in the *SlMYB12* promoter, a splicing disruption in *SlSGR1*, and a retrotransposon insertion in *SlPSY1*, leading to green ripe fruit in Lvbaoshi tomato. The study provides novel insights into a unique regulatory mechanism that governs fruit color in both cultivated and wild tomato species.**

Tomato (*Solanum lycopersicum*) fruit coloration, primarily determined by pigments such as carotenoids and flavonoids, is a complex trait influenced by multiple genetic factors. Tomato, among the most widely consumed fruits globally, exhibits a spectrum of colors ranging from green to red (Yang *et al*., 2019, 2023). Understanding the genetic mechanisms governing fruit color transition in tomato is crucial for agricultural breeding programs, and sheds light on evolutionary aspects. The quest for enhancing tomato quality has led breeders to focus on traits beyond mere yield and storage properties, with fruit color being a pivotal consideration (Kilambi *et al*., 2017; Tieman *et al*., 2017). Fruit color, dynamically changing during ripening, holds both aesthetic appeal for consumers and biological significance in the maturation process (Yang *et al*., 2019). Various pigments, including chlorophylls, carotenoids, and flavonoids, contribute to the diverse colors observed in ripe tomato fruit (Gao *et al*., 2015). Through the isolation of mutant strains, researchers have delineated the crucial role of genes in the carotenoid biosynthesis pathway, leading to fruit colors ranging from green to purple, orange/yellow, and pink (Dhar *et al*., 2015; Gao *et al*., 2016). The enzyme phytoene synthase (*PSY1*), a key regulator in carotenoid biosynthesis, catalyzes the formation of phytoene. Mutations in *PSY1* result in distinct fruit phenotypes such as pale-yellow flesh or yellow skin (Fray and Grierson, 1993; Kachanovsky *et al*., 2012). Similarly, the enzyme carotene *cis-trans* isomerase (CRTISO) plays a crucial role in determining fruit color, with mutations resulting in the accumulation of prolycopene instead of all-*trans*-lycopene, leading to orange-colored fruit (Kachanovsky *et al*., 2012). In red-fruited tomatoes, enzymes involved in flavonoid biosynthesis, such as chalcone synthase (CHS), flavanone 3-hydrolase (F3H), and flavonol synthase (FLS), play pivotal roles (Wang *et al*., 2021). Furthermore, regulatory genes such as *MYB12*, governing *CHS* expression, have been identified as contributory factors for specific fruit colors (Ballester *et al*., 2010).

## Genetic basis of green ripe fruit

The transition from green to red ripe fruit during tomato ripening is a complex process, which is governed by the conversion of chloroplasts to chromoplasts, accompanied by changes in pigment composition and accumulation (Gao *et al*., 2015; Kilambi *et al*., 2017; Sharma *et al*., 2023). Recent advances have shed light on the roles of specific genes in tomato fruit coloration, including *SGR1* (*STAY-GREEN 1*), *RIN* (*RIPENING INHIBITOR*), *CNR* (*COLORLESS NON-RIPENING*), and *NOR* (*NON-RIPENING*) (Zhu *et al*., 2014). Notably, *SGR1* has been implicated in the regulation of chlorophyll degradation, with its suppression resulting in the accumulation of chlorophylls and carotenoids in ripe fruit (Luo *et al*., 2013). Despite significant advances in understanding the genetic basis of tomato fruit coloration, the mechanisms underlying uncommon ripe fruit colors, such as green, remain elusive. The domestication of tomato has led to the prevalence of red or pink ripe fruit in cultivated species, while most wild tomato exhibit green-colored fruit at maturity (Ballester *et al*., 2010). The study by Cui *et al*. (2024) stands out for its innovative approach to unravel the genetic basis of green ripe fruit in tomato ‘Lvbaoshi’ (LBS) cultivar, which is known for its green ripe fruit with colorless epidermis and green fresh. By employing a combination of genetic mapping, bulk segregant analysis, RNA sequencing, and transgenic experiments, Cui *et al*. characterized the green ripe fruit phenotype of the LBS cultivar, attributing it to mutations in three distinct loci: *SlSGR1*, *SlPSY1*, and *SlMYB12*. A single nucleotide (T/C) substitution in *SlSGR1* results in disruption of mRNA splicing and truncation of the translated protein, eventually leading to the retention of chlorophyll and the manifestation of green fruit color during ripening. This finding aligns with previous studies highlighting the role of *SGR1* in fruit color regulation (Barry *et al*., 2008; Barry and Pandey, 2009). Similarly, mutations in *SlPSY1*, encoding phytoene synthase 1, contribute to green ripe fruit in LBS. A retrotransposon insertion in the first exon of *SlPSY1* hampers its transcription, impeding carotenoid biosynthesis and resulting in green fruit coloration at ripening. These results are consistent with studies suggesting the involvement of *PSY1* in carotenoid accumulation and fruit coloration (Bolger *et al*., 2014; Kilambi *et al*., 2017). Furthermore, a 603 bp deletion in the promoter region of *SlMYB12*, a transcription factor governing fruit pigmentation, was linked to the colorless peel phenotype observed in LBS. This deletion disrupts *MYB12* expression, inhibiting the synthesis of pigments responsible for fruit coloration. These findings are consistent with previous research indicating that *MYB12* plays a crucial role in regulating fruit color development (Ballester *et al*., 2010; Lin *et al*., 2014).

## Insights from wild tomato species

The investigation extended to wild tomato species bearing green ripe fruit, shedding light on their genetic mechanisms. Transgenic experiments revealed that the *SGR1* gene from wild tomato *Solanum pennellii* may harbor mutations impairing its function, contributing to the prevalence of green fruit phenotypes. Similarly, variations in the *PSY1* gene of *Solanum habrochaites* and *S. pennellii* were associated with reduced PSY1 protein levels, further elucidating the genetic basis of green fruit coloration in wild relatives. The simultaneous suppression of *SGR1* and *PSY1* by RNAi in a pink fruit cultivar transformed the pink fruit into green ripe fruit in RNAi plants, suggesting that the green ripe fruit trait of LBS is due to down-regulation of SGR1 and PSY1 proteins. Moreover, the green or gray green ripe fruit of many wild tomato species is partially due to a single amino acid change in PSY1 and a deletion in the promoter of *SGR1*. However, transgenic lines of the purple flesh *green-flesh* (*gf*) mutant expressing *ProSGR1::SGR1-PNL* from *S. pennellii* failed to convert the purple-flesh fruit into the red-flesh fruit. Moreover, overexpression of *PSY1* of *S. pennellii* in LBS plants partially restored the green ripe fruit phenotype, though it was not fully rescued. Collectively, these findings suggest that the *SGR1* gene in *S. pennellii* may be functionally impaired, leading to green ripe fruit. The reduced abundance of the PSY1 protein in *S. habrochaites* and *S. pennellii* also contributes to the green ripe fruit trait in wild tomato species.

**Fig. 1.**
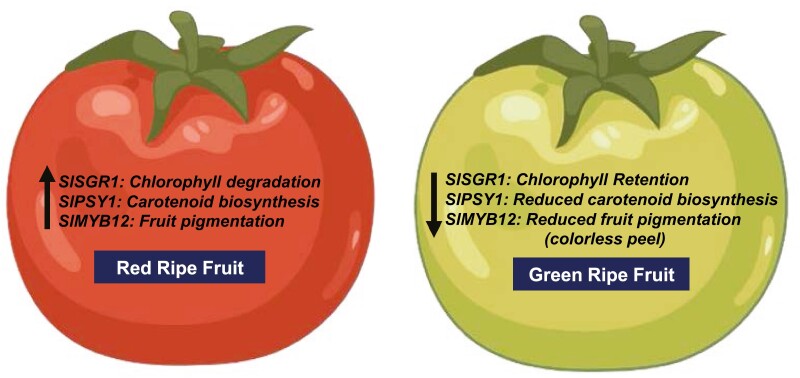
Down-regulated expression of *SGR1*, *PSY1*, and *MYB12* genes results in green ripe fruit in cultivated and wild tomatoes (created with BioRender.com).

The study also emphasized the role of the lycopene biosynthesis pathway in green fruit formation. Disruptions in lycopene biosynthesis pathway enzymes such as PSY1 and overexpression of lycopene β-cyclase (CYC-B) were shown to impede carotenoid metabolism, resulting in green ripe fruit. Other enzymes of lycopene pathways also offer an opportunity to understand the green ripe fruit production. These underscore the significance of the lycopene metabolic pathway in determining fruit color and highlight potential targets for breeding programs aimed at modulating fruit pigmentation. Beyond enzyme mutations, the role of disruptions in regulatory factors such as the *RIN* gene were implicated in green fruit development. The CRISPR/Cas9-mediated knockout of *RIN* in *gf* plants resulted in the development of green ripe fruit, highlighting that the intricate regulatory network governing fruit color and ripening exists. Taken together, all these insights expand our understanding of the multifaceted mechanisms underlying fruit coloration in tomato. This work uncovers a novel regulatory mechanism by which *MYB12*, *PSY1*, and *SGR1* regulate fruit color in cultivated green ripe tomato and some wild tomato species (Fig. 1).

## Implications for breeding and beyond

The findings of this study not only deepen the understanding of tomato color regulation but also pose intriguing questions for future research. The observed variations in gene sequences among wild and cultivated tomato hint at a rich reservoir of genetic diversity. Exploring these variations can potentially lead to the development of tomato cultivars with novel colors and enhanced nutritional profiles. The identification of specific genetic variants associated with green ripe fruit offers opportunities for marker-assisted selection and the development of novel tomato varieties with improved traits. Furthermore, the findings highlight the complexity of fruit ripening regulation and its relevance beyond tomato. The parallels drawn between tomato mutants and wild relatives, such as *S. pennellii*, underscore the evolutionary significance of these genetic pathways and their potential adaptation to diverse environmental conditions.
